# Cytoskeletal dysregulation and neurodegenerative disease: Formation, monitoring, and inhibition of cofilin-actin rods

**DOI:** 10.3389/fncel.2022.982074

**Published:** 2022-09-22

**Authors:** Anna I. Wurz, Anna M. Schulz, Collin T. O’Bryant, Josephine F. Sharp, Robert M. Hughes

**Affiliations:** ^1^Department of Chemistry, East Carolina University, Greenville, NC, United States; ^2^Department of Chemistry, Notre Dame College, South Euclid, OH, United States

**Keywords:** cofilin, actin, cytoplasmic rods, nuclear rods, stress, neurodegenerative disease, Alzheimer’s disease, cytoskeletal dysregulation

## Abstract

The presence of atypical cytoskeletal dynamics, structures, and associated morphologies is a common theme uniting numerous diseases and developmental disorders. In particular, cytoskeletal dysregulation is a common cellular feature of Alzheimer’s disease, Parkinson’s disease, and Huntington’s disease. While the numerous activators and inhibitors of dysregulation present complexities for characterizing these elements as byproducts or initiators of the disease state, it is increasingly clear that a better understanding of these anomalies is critical for advancing the state of knowledge and plan of therapeutic attack. In this review, we focus on the hallmarks of cytoskeletal dysregulation that are associated with cofilin-linked actin regulation, with a particular emphasis on the formation, monitoring, and inhibition of cofilin-actin rods. We also review actin-associated proteins other than cofilin with links to cytoskeleton-associated neurodegenerative processes, recognizing that cofilin-actin rods comprise one strand of a vast web of interactions that occur as a result of cytoskeletal dysregulation. Our aim is to present a current perspective on cytoskeletal dysregulation, connecting recent developments in our understanding with emerging strategies for biosensing and biomimicry that will help shape future directions of the field.

## Introduction

Oxidative and energetic stress are primary progenitors of human disease (Kregel and Zhang, 2007; Pizzino et al., 2017). In the context of Alzheimer’s disease (AD) and other neurodegenerative disorders, the presence of these stressors can initiate cellular processes leading to synapse decay and eventual impaired neural function (Chen and Wang, 2015; Alsegiani and Shah, 2020). Imbalances in stress-inducing molecules promote cytoskeletal dysregulation in cells; in particular, neurons undergoing stress form persistent rod-like structures that contain primarily cofilin and actin (hence the term ‘cofilin-actin rods’) (Bamburg et al., 2010). A growing body of evidence suggests that rods play an important role in neurodegeneration, both as indicators of early-stage disease and as a potential therapeutic target (Jang et al., 2005). Persistent cofilin-actin rods are thought to interfere with transport in synapses, altering cytoskeletal dynamics and interrupting cell-to-cell communication (Maloney et al., 2005; Maloney and Bamburg, 2007; Bamburg et al., 2010). As the cytoskeleton is a critical component of learning and memory, cofilin-actin rod-associated alterations in its normal function present one possible pathway to the disease state.

Increasingly, neurodegenerative diseases are linked to defects in synaptic plasticity, or the ability of synapses to reconfigure over time in response to external cues (Bliss et al., 2014). Diseases in which synaptic plasticity and associated dendritic spine defects are pronounced include AD, PD, HD, SCZ, and ASD (Borovac et al., 2018). Dendritic spines are a focal point of synaptic function as they govern both synaptic excitatory and inhibitory transmission, with spine number, density, and distribution contributing to synaptic function (Ben Zablah et al., 2020; Lamprecht, 2021). Actin is a primary component of dendritic spines, making actin dynamics critical for their structural plasticity (Borovac et al., 2018). In addition, the actin binding protein cofilin comprises a critical component of spine maintenance (Bosch et al., 2014). Cofilin has been called a “master node” in the pathogenesis of Alzheimer’s disease and is commonly found in the brain tissue of AD patients in the form of cofilin-actin rods (Kang and Woo, 2019). However, despite the association of cofilin-actin rods with the AD brain, the precise role of cofilin-actin rods in neurodegenerative disease remains an open question. For instance, rods have been described as protective in the short term by delaying accumulation of actin-cofilin at the outer mitochondrial membrane with subsequent cytochrome C release and downstream apoptosis (Bernstein et al., 2006). Alternately, the long-term presence of persistent cofilin-actin rods is thought to inhibit fundamental intracellular transport processes, serve as an accumulation site for APP, and play a role in tau accumulation and fibril formation (Whiteman et al., 2009, 2011). Rods have also been proposed to eventually mature into Hirano bodies (Minamide et al., 2000), large actin inclusions that are found in Alzheimer’s and other neurodegenerative diseases (Spears et al., 2014; Kang and Woo, 2019). Thus, the intriguing, and possibly conflicting, roles of cofilin-actin rods invite further investigation (Bamburg et al., 2010; Bamburg and Bernstein, 2016; Kang and Woo, 2019; Wang Q. et al., 2020).

Pertinently, the monitoring of cofilin-actin rods in living cells and organisms remains a challenge. While both Lifeact, a short polypeptide with affinity for F-actin, and phalloidin, a bicyclic heptapeptide with high F-actin affinity, have widespread applications in the monitoring of actin-rich structures in cells (Mazloom-Farsibaf et al., 2021), they both have limitations in the detection of cofilin-actin rods. Lifeact does not bind to cofilin-actin rods (Munsie et al., 2009), and fluorescent phalloidin does not stain, or only weakly stains in some cases, cofilin-actin rods (Bamburg et al., 2021). As a result, two of the most potent options for visualizing actin are unavailable for the study of cofilin-actin rods. This highlights the need for novel strategies enabling the monitoring of rod formation and measurement of rod dynamics. As such, in this review, while we focus on the hallmarks of cytoskeletal dysregulation that are associated with cofilin-linked actin regulation, we place a particular emphasis on current methodologies for the monitoring and inhibition of cofilin-actin rods.

## Initiators, promoters, and effects of cofilin-actin rod formation

**FIGURE 1:**
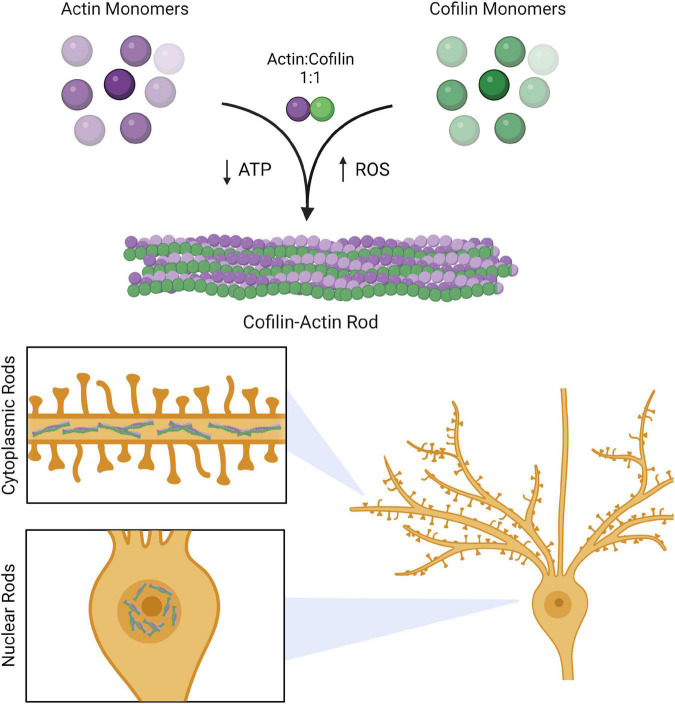
Cofilin-actin rods in neurons. Actin and cofilin bind to each other in a 1:1 ratio. Persistent cofilin-actin rods can occur under stress conditions, such as ATP depletion or oxidative stress brought upon by ROS. In neurons, these rods accumulate in the cytoplasm of processes (cytoplasmic rods) and in the nucleus (nuclear rods). Created with www.biorender.com.

Cofilin-actin rods are largely comprised of dephosphorylated (active) cofilin and ADP-actin in a 1:1 ratio (Figure 1) (Minamide et al., 2010). Several stress-responsive cell signaling pathways lead to rod formation (Bamburg et al., 2010). In the context of AD, cofilin dephosphorylation occurs as a result of Aβ activation of the Cdc42 pathway leading to subsequent downregulation of RhoA and LIMK (Chen and Wang, 2015), while ATP depletion during oxidative stress promotes the ADP-bound actin state (Atkinson et al., 2004). Rods contain a highly twisted form of actin that is decorated with bound cofilin molecules (McGough et al., 1997; Bernstein et al., 2012). Critically, while rods presumably bear some similarities to the actin-cofilin interface present under conditions of homeostasis, there are currently no available crystal structures of cofilin-actin rods that could provide critical structural insights. As a result, many insights into cofilin-actin rod structure have come from site directed mutagenesis experiments. For example, mutagenesis has identified intramolecular cofilin oxidation (C147–C139 and C80–C39) as an important component of rod formation (Bernstein et al., 2012). In addition, while other proteins have been identified in cofilin-actin rods, e.g., 14-3-3 in cytoplasmic rods (Minamide et al., 2010) and coronin and filactin in nuclear rods (Ishikawa-Ankerhold et al., 2017), cofilin and actin are the primary drivers of rod formation (Minamide et al., 2010).

**FIGURE 2:**
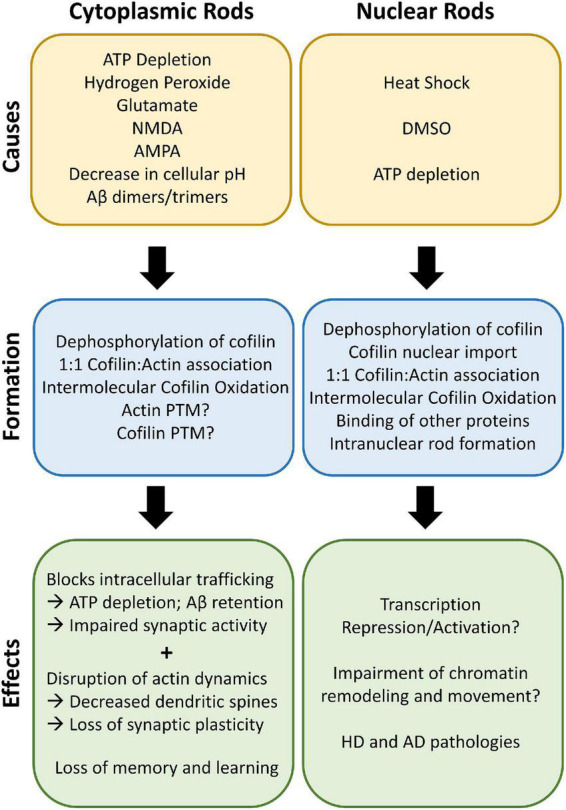
Cofilin-actin rod causes and effects. Cytoplasmic rods can be induced by multiple types of stress, such as ATP depletion, oxidative stress, and a decrease in cellular pH. Dephosphorylation of cofilin and association with actin in a 1:1 ratio leads to rod formation in the presence of oxidative stress. Post-translational modifications (PTM) of actin and cofilin may also be an emerging area for rod regulation. The rods block critical intracellular trafficking of organelles such as mitochondria and results in ATP depletion and impaired synaptic activity. The disruption of actin dynamics due to sequestered cofilin decreases dendritic spines and loss of synaptic plasticity, leading to loss of memory and cognitive ability over time. Interestingly, nuclear rods are similarly formed in the nucleus after nuclear translocation due to heat shock, DMSO, or ATP depletion. Other actin-binding proteins (ABPs), including the Huntingtin mutant, associate with the nuclear rods and form persistent rods that can affect transcription and chromatin remodeling. Both cytoplasmic and nuclear rods can lead to AD, HD, and PD pathologies.

Rod formation can be initiated with a wide array of stress-associated stimuli; as the field of cofilin-actin rod research has developed, many of these stimuli and their associated signaling pathways have been identified and compiled (Minamide et al., 2000; Kang and Woo, 2019; Ben Zablah et al., 2020). Here we highlight both the promoters and initiators of rod formation (see Section “Initiators and promoters of cofilin-actin rods”) and the downstream impacts of cofilin-actin rods (see Section “Effects of cofilin-actin rods”; summarized in Figure 2).

### Initiators and promoters of cofilin-actin rods

#### Oxidative and energetic stress promoters and glutamate excitotoxicity

Various promoters of oxidative and energetic stress are known to induce cofilin-actin rod formation in cultured cells (Minamide et al., 2000). In cultured hippocampal neurons, ATP depletion (10 mM NaN_3_ and 6 mM 2-DG/30 min), hydrogen peroxide (10 μM/60 min), and glutamate (150 – 300 μM/30 min) have demonstrated efficacy in cofilin-actin rod induction (Minamide et al., 2000). In hippocampal neurons, stimulation of AMPA receptors (AMPAR) by excess glutamate mediates Ca^2+^-dependent rod formation (Bamburg et al., 2010). Often, neurodegenerative stimuli are also effective at inducing cofilin-actin rods in immortalized cells such as HeLa and HEK 293 (Minamide et al., 2000; Salem et al., 2020). As a result, these immortalized cell lines are useful in proof-of-principle experiments prior to more biologically relevant investigations in neuronal culture.

#### Metabolic stress and changes in cellular pH

*Dictyostelium discodieum*, commonly used to study actin dynamics (Aizawa et al., 1995; Khamviwath et al., 2013; Ishikawa-Ankerhold et al., 2017), has been used recently to study cytoplasmic cofilin-actin rod formation (Ishikawa-Ankerhold et al., 2021). ATP depletion was induced using sodium azide, dinitrophenol, or 2-deoxyglucose treatment; as anticipated, these treatments produced cofilin-actin rods that were reversible upon restoration of non-ATP depleting media. Interestingly, hyperosmotic shock (200 or 400 mM sorbitol) also induced cofilin actin rod formation and was accompanied by a drop in intracellular pH (Ishikawa-Ankerhold et al., 2021). Since the reduction of cellular pH was responsible for activating cofilin-actin rod formation, it was further hypothesized that intracellular pH changes induced by aberrant phase transition processes could initiate, at least transiently, cofilin-actin rod formation (Ishikawa-Ankerhold et al., 2021). These results may provide a mechanistic link between pH regulated phase separation and cytoskeleton-associated disease progression.

#### Slingshot homolog-1 and LIM kinase regulate cofilin

**FIGURE 3:**
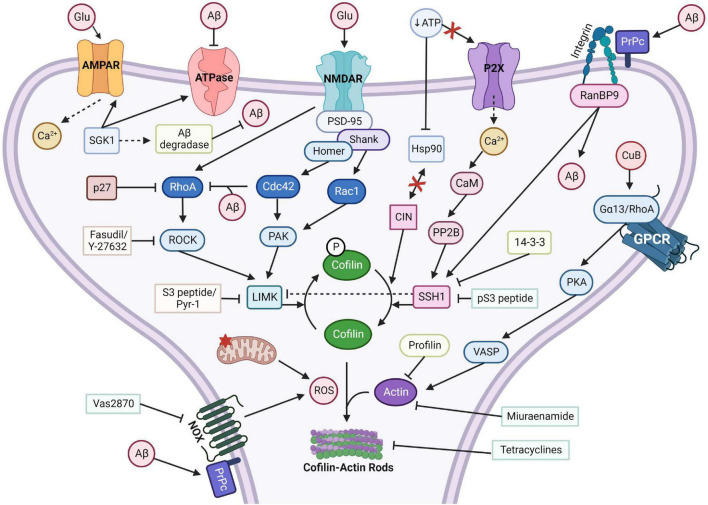
Cofilin-actin rod forming pathways with activators and inhibitors. Cofilin-actin rods can be induced through multiple separate pathways. Calcineurin (PP2B) or RanBP9 stimulation of SSH1 or chronophin (CIN) dephosphorylates and activates cofilin, which under stress can form aberrant rods with actin. Inhibitors of SSH1, such as 14-3-3 and pS3 peptide, can prevent rod formation. Miuraenamide and profilin overexpression can prevent actin from associating with cofilin, and tetracycline can disrupt rods. Aβ induction of integrin/RanBP9 or NOX with PrPc promotes rod formation, but Vas2870 can inhibit NOX generation of ROS. CuB activates the Gα13/RhoA/VASP pathway and increases actin assembly. LIMK, activated by PAK and RhoA/ROCK, phosphorylates and inactivates cofilin. Inhibitors of LIMK, such as the S3 peptide or Pyr-1, can promote cofilin-actin rod formation. Created with www.biorender.com.

Cofilin is inactivated via phosphorylation of Serine 3 by the serine/threonine protein kinase, LIM-domain kinase (LIMK) (Ben Zablah et al., 2021), and can be reactivated via dephosphorylation by SSH1 phosphatase (Soosairajah et al., 2005). Both LIMK1 and LIMK2 can inactivate the actin depolymerization ability of cofilin, halting active actin dynamics temporally (Ben Zablah et al., 2021). Upstream activators of LIMK are the Rho GTPases, RhoA, Rac1, and Cdc42, in conjunction with their kinase effectors, ROCK and p21-activated kinase (PAK1/4) (Edwards et al., 1999). In addition to acting on ADF/cofilin, SSH1 can inactivate LIMK via direct dephosphorylation (Soosairajah et al., 2005). LIMK1 also interacts with 14-3-3 scaffold proteins and as a result is dephosphorylated and downregulated (Soosairajah et al., 2005). In fibroblasts with Rac1 upregulation, LIMK associates with either SSH1 or 14-3-3z and localizes to growth cones and membrane ruffles; *in vitro*, all three proteins have been shown to interact with each other. Furthermore, SSH1 activity is inhibited by PAK4 phosphorylation (Soosairajah et al., 2005). For a visual depiction of complex signaling interplay required for cofilin regulation, see Figure 3.

#### CDC42 promotes cofilin-actin rod formation

Cdc42, a Rho family GTPase that regulates the activity of cofilin, has been linked to the formation of cofilin-actin rods induced by Aβ oligomer-related stress (Davis et al., 2009). Davis et al. (2009) studied the effect of Cdc42 activity in neurons treated with Aβ_1–42_ oligomers. For neurons that overexpressed wildtype or constitutively active Cdc42 in the absence of Aβ oligomers, the percentage of neurons that formed rods increased 10-fold compared to control neurons (Davis et al., 2009). Knockdown of Cdc42 resulted in rod levels similar to control neurons. In the presence of Aβ oligomers, 19% of control neurons formed rods, whereas a significant increase in the percentage (30%) of rod-forming cells was observed in the constitutively active Cdc42 neurons. Only 7.5% of the Cdc42 knockdown neurons produced rods under Aβ stress. These results demonstrate that while Aβ induces cofilin-actin rod formation through multiple pathways, at least one of them is dependent on Cdc42 (Davis et al., 2009).

#### CKS1/p27

Cyclin-dependent kinase regulatory subunit 1 (CKS1) is a protein that interacts with cyclin-dependent kinases and regulates entry into the S-phase during the cell cycle (Pines, 1996). CKS1 regulates the phosphorylation of cofilin which enables actin cytoskeleton turnover, a key process in the maturation of dendritic spines. The absence of CKS1 compromises the establishment of long-term potentiation (LTP) and the functionality of dendritic synapses (Kukalev et al., 2017). Because of this critical role in neurons, CKS1 is implicated in long-term memory formation in the adult brain. A related protein, p27, inhibits RhoA, an upstream regulator of cofilin phosphorylation, promoting cofilin activation and cofilin-actin rod formation (Kukalev et al., 2017). As CKS1 has been shown to modulate p27 degradation (Spruck et al., 2001), CKS1 knockdown resulted in p27 inhibition of RhoA, causing an increase in dephosphorylated cofilin and eventual cofilin-actin rod formation. Overall, CKS1 is a key contributing factor in memory protection, LTP, and cell dendritic spine maturation within the adult hippocampus (Kukalev et al., 2017).

#### Profilin 2 mediates cofilin-actin rod formation

Cofilin-actin rods have been observed in not only neurodegenerative disease, but also in neuromuscular disease, specifically SMA (Rademacher et al., 2017). This autosomal recessive disease afflicting children is commonly caused by a loss-of-function mutation of the gene encoding the protein Survival of Motoneuron (Keinath et al., 2021). The actin-binding protein, Profilin2, has been identified as a key protein within rod formation in a model of SMA (Walter et al., 2021). Dysregulation of RhoA and ROCK induces hyperphosphorylation of profilin2 via its Ser137 residue. Profilin then binds to and actively promotes cofilin-actin rod formation through its actin bundling ability (Walter et al., 2021). Rescuing the functionality of the RhoA/ROCK pathway could alleviate the symptoms of SMA caused by actin rods.

#### p38^MAPK^-M2 signaling axis

The p38^MAPK^-MK2 signaling axis is linked to cofilin activity through the p38^MAPK^/MK2/LIMK1/cofilin signaling cascade (Beamer and Corrêa, 2021). Within neuronal synapses, the MAPK protein p38 is stimulated by various inflammation signaling pathways: RAGE/p21^Ras^ (Yeh et al., 2001); IL1R/MyD88/IRAK (Dunne and O’Neill, 2003); TGFβR/TAK1 (Yu et al., 2002); and TNF1/TRAF2 (Shi and Sun, 2018). MK2 is a downstream phosphorylation target for p38 and regulates LIMK1 phosphorylation of cofilin (Kobayashi et al., 2006). p38^MAPK^-MK2 signaling axis additionally modulates synaptic neurotransmission after the inflammatory response (Beamer and Corrêa, 2021). These pathways provide a potential link between synaptic plasticity in response to inflammatory stress stimuli and the dysregulation of cofilin leading to cofilin-actin rod formation.

#### Endothelin type B receptor activation

Overexpression of endothelin-1 (ET-1), a neuroactive peptide, has been shown to induce cofilin-actin rods in primary hippocampal neurons (Tam et al., 2019). There is a direct connection between ET-1 and AD, as elevated levels of this peptide have been identified in post-mortem brains of AD patients (Minami et al., 1995). Tam et al. (2019) showed that ET-1-dependent activation of the ET_*B*_ induced cofilin oxidation via the NOX pathway and cofilin activation via the Calcineurin/SSH1 pathway, leading to increased rod formation, blockage of intracellular trafficking, synapse loss, and eventual dendritic loss.

#### Prion-protein dependent pathway

Cellular prion protein (PrPc), a cell surface protein expressed in the central and peripheral nervous system (Wulf et al., 2017), interacts directly with Aβ and is implicated in cofilin-actin rod formation associated with AD (Walsh et al., 2014a). Walsh et al. (2014a) investigated the role of PrPc during Aβ oligomer and pro-inflammatory cytokine (TNFα, IL-1β, and IL-6) induction of rod formation in rat hippocampal neurons. These stimuli individually induced rods in about 20% of neurons and resulted in an increase of dephosphorylated cofilin near areas of rod accumulation. In PrPc-null neurons, the number of neurons exhibiting rod formation significantly decreased, and rod formation in these neurons was rescued upon adenovirus-mediated expression of PrPc. By contrast, glutamate and ATP-depletion-induced rods were not dependent on PrPc expression. Studies using NOX knockdown and NOX inhibitors (TG6-227, ML171, apocynin, and DNp22) further demonstrated that NOX activity is required for rod formation in the presence of Aβ and TNFα (Walsh et al., 2014b). Additionally, an overexpression of PrPc induces rod formation comparable to the amount of rod formation in Aβ and TNFα-stimulated neurons. PrPc-induced rod formation was also dependent on NOX pathway activation (Walsh et al., 2014b). Based on these results, a proposed mechanism for rod formation details concentrated PrPc at the extracellular plasma membrane stimulating NOX activity, resulting in intracellular reactive oxygen species (ROS) generation and cofilin-actin rod induction. Identification of a common PrPc/NOX-dependent pathway for multiple disease-associated agents (Aβ, cytokines) makes PrPc/NOX an attractive therapeutic target (Walsh et al., 2014a).

#### Alpha-synuclein/prion in Parkinson’s disease

Aggregated α-syn is a hallmark of PD and disrupts the structure and dynamics of the actin cytoskeleton (Esposito et al., 2007; Sousa et al., 2009; Brás et al., 2018). As PrPc can interact with Aβ oligomers in AD pathology (Walsh et al., 2014a), the role of PrPc in cell-to-cell spreading of α-syn pathology and subsequent cognitive deficits have been explored (Esposito et al., 2007; Sousa et al., 2009; Brás et al., 2018). *In vitro* studies showed that the N-terminal domain of PrPc directly interacts with α-syn fibrils similar to Aβ oligomers (Aulić et al., 2017; Ferreira et al., 2017; Brás et al., 2018), and the expression of PrPc significantly increased the uptake of α-syn amyloid fibrils in primary hippocampal neurons (Aulić et al., 2017). *In vivo*, PrPc-null mice exhibited less accumulation of α-syn aggregates throughout the brain with no accumulation in the striatum (Aulić et al., 2017). Further elucidation of this signaling pathway revealed that the synaptic transmembrane protein, mGluR5, is a mediator between α-syn-PrPc and the activation Fyn, a Src tyrosine kinase. Fyn subsequently activates NMDA receptor (NMDAR) subunit B leading to calcium dysregulation, LTP inhibition, and synaptic dysfunction (Ferreira et al., 2017; Brás et al., 2018). As a result of NMDAR activation, an increase in intracellular calcium activates calmodulin and calcineurin, leading to cofilin dephosphorylation through SSH1 activity. The activated cofilin from this pathway has been proposed to form cofilin-actin rods (Ferreira et al., 2017; Brás et al., 2018); however, a direct connection between the α-syn/PrPc signaling pathway and cofilin-actin rod formation has yet to be determined (Ferreira et al., 2017; Brás et al., 2018).

#### Cofilin-actin rods induced by ischemia

Cofilin has been implicated in neuronal damage during ischemia (Hoffmann et al., 2019), a condition that reduces blood flow to the brain eventually causing neuron death and permanent brain damage (Madineni et al., 2016). During ischemic stroke, cofilin-actin rods have been shown to impair dendritic spines, reduce vesicle and organelle movement to synapses, and induce synaptic loss, particularly in the peri-infarct region. Dephosphorylated cofilin and rod formation upon cellular stress from ischemic stroke have yielded effects similar to the neuronal impairment described above. In mice models of ischemic stroke, LIMK1 overexpression has potential to reduce the number of rods after energy depletion. Therapeutics that preserve cofilin phosphorylation could rescue synaptic loss and prevent neuronal damage after ischemic stroke. As ischemic responses have been demonstrated to be initially localized to the neuronal cytoskeleton, cofilin could be involved in early apoptosis of neurons during ischemia. To study this, PC12 cell and mouse primary cortical neurons were analyzed for cofilin expression patterns (Madineni et al., 2016). Additionally, two *in vivo* mouse models of ischemia were utilized: chemically induced oxidative stress and oxygen-glucose deprivation/reperfusion. The expression profile of each model of ischemia showed a reduction in p-cofilin, suggesting an activation of cofilin in response to stress. Furthermore, the results suggested that the phosphatases calcineurin and SSH1 are signaling mediators of cofilin dephosphorylation. Knockdown of cofilin by siRNA resulted in decreased cofilin levels in the control and ischemia models, correlating to reduced cofilin-apoptosis pathways and increased neuron viability (Madineni et al., 2016). Results provide a correlation between cofilin’s role in apoptosis and ischemia-induced neuronal degradation with a potential for therapeutic targets. *Rod formation after brain ischemia:* The severity of ischemic stroke varies depending on the time frame after onset and the extent to which reperfusion occurs that can preserve viability of tissue (Won et al., 2018). Won et al. (2018) experimented with varying courses of time and location of cofilin rod formation in different models, three of which included reperfusion at various times and locals with a fourth exhibiting permanent ischemia (no reperfusion). Both the region and relative interval of ischemia were found to be instrumental in the persistence of cofilin rod formation and survival (Won et al., 2018). Upon reperfusion after a brief ischemic event, rod formation increased between 0 and 4 h after reperfusion, depending on type of ischemia and location of tissue analysis, and then significantly decreased after 24 h. As rod formation can be induced by oxidative stress, the increase in rods after reperfusion correlates to the oxidative stress that occurs as oxygenated blood flow is reintroduced to the tissue. The models of permanent ischemia and transient focal ischemia showed an increase in rod formation over the full 24 h; interestingly, rod formation was observed within adjacent non-ischemic tissue in the permanent ischemia model (Won et al., 2018). Interval of ischemia and the start time of reperfusion after ischemia should be considered as essential factors to ischemic stroke treatment depending on the location and reversibility of neurite damage.

#### Herpes simplex virus type 1 dysregulation of cofilin

Herpes simplex virus type 1 (HSV-1), a neuroinvasive virus, has been linked to moderate cognitive impairment in recent population-based studies (Murphy et al., 2021) and has also been associated with the development and poor prognosis of AD in patients with comorbidity (Itzhaki et al., 1997; Wang Y. et al., 2020). While not conclusive evidence, HSV-1 DNA has been found to colocalize with Aβ plaques in human AD brains (Wozniak et al., 2009) and promote Aβ accumulation in neurons (Wozniak et al., 2007). Connections between HSV-1 infection and cofilin-associated cytoskeletal dysregulation and subsequent loss of synaptic plasticity have been proposed (Wang Y. et al., 2020). When HSV-1 infects a host cell, actin dynamics are manipulated via LIMK and SSH1 regulation of cofilin during the viral lifecycle (Zheng et al., 2016). HSV-1 alone causes cofilin dysregulation with subsequent memory and cognitive defects (Zheng et al., 2016; Murphy et al., 2021). Coupled with AD, HSV-1 exacerbates this deteriorating cognitive ability (Wang Y. et al., 2020). Further studies exploring the underlying mechanisms influencing this pathology are needed.

#### Human immunodeficiency virus infection leads to rod formation

Human immunodeficiency virus (HIV) infection in the CNS leads to increased production of inflammatory and neurotoxic substances which results in an occurring degradation of the synapses along with overall cognitive decline (Smith et al., 2021). The glycoprotein gp120, present in the HIV viral envelope, has been shown to induce cofilin-actin rods within cultured mouse hippocampal neurons (Smith et al., 2021). The formation of these rods is caused by gp120 entry of the host cell at the host cell lipid raft domains, followed by gp120 binding and activation of chemokine receptors CCR5 and CXCR4. This pathway along with gp120 interaction with membrane-bound PrPc leads to NOX activation and ROS generation. SSH1 activation is stimulated by the oxidative environment and dephosphorylates cofilin, resulting in cofilin-actin rod formation. The synaptic damage caused by cofilin-actin rods could be an etiology of HIV-associated neurocognitive disorders (HAND) presented in long-term HIV-afflicted individuals (Smith et al., 2021).

#### Nuclear rods and cofilin nuclear-cytoplasmic shuttling

Cofilin has a bipartite nuclear localization sequence (NLS) and CRM1-dependent nuclear export sequence (NES). As a result, a significant amount of cofilin can accumulate in the nucleus during stress and form nuclear cofilin-actin rods (Munsie et al., 2012). Shuttling of cofilin in and out of the nucleus during stress may be a critical part of this stress response. Two point mutants to the bipartite NLS were utilized to study actin binding and nuclear translocation. K22A and R21A both bind actin *in vivo* and affect nuclear localization; however, K22A has the ability to form internuclear rods with actin and R21A does not (Munsie et al., 2012). Both mutants have applications in further defining the role of nuclear cofilin shuttling in the cofilin-actin rod stress response with a potential for site-directed gene therapy targeting the cofilin transcriptome.

#### Promoters of nuclear cofilin-actin rod formation

DMSO or heat shock treatment induces intranuclear cofilin-actin rods in mammalian cells (Munsie et al., 2011; Kelpsch and Tootle, 2018). Munsie et al. (2011) demonstrated that the actin-binding protein huntingtin is required for normal formation and clearance of nuclear rods. However, in the presence of mutant huntingtin protein, rod formation and persistence are altered, resulting in fewer, but longer lasting rods per cell. This provides a mechanism for rod-associated pathogenesis in HD (Atwal et al., 2007). Rod persistence is associated with transglutaminase 2 (TG2) activity, which is activated by stress-induced calcium release from the ER and subsequently crosslinks actin and cofilin (Munsie et al., 2011). The impacts of nuclear rods on gene expression are also of interest. Under normal conditions, nuclear cofilin and actin play a role in mRNA transcription through interaction with RNA Polymerase II (Obrdlik and Percipalle, 2011), and nuclear actin additionally regulates chromatin remodeling and nuclear structure; therefore, the accumulation of nuclear rods could impair gene expression and these other processes (Dopie et al., 2012; Kelpsch and Tootle, 2018).

#### Nuclear rods and heat stress in Drosophila embryos

Figard et al. (2019) demonstrate an analogous actin stress response in *Drosophila* embryos to that previously characterized in mammalian cells: heat stress triggers cofilin-actin rod assembly in the nucleus. Specifically, intranuclear rod formation was observed at 32°C alongside an increase in dephosphorylated active cofilin. The intranuclear rods disassembled when embryos were removed from heat stress. At furrow tips, the active cofilin destabilized the cytoplasmic F-actin integral to morphogenesis, freeing actin monomers to migrate to the nucleus, sequester, and form rods. Heat-shocked embryos were then incubated at a lower temperature and assessed for viability. The destabilization of F-actin at furrow tips reduced wildtype embryo viability by about 12%, whereas embryos expressing a lower level of cofilin (*cofilin*^±^) had a decrease in viability by about 4%. Interestingly, the wildtype and reduced cofilin embryos exhibited the same percentage of multinucleation as a result of furrow regression, suggesting that viability correlated with F-actin stabilization and not morphogenetic defects. Overall, F-actin stabilization during heat shock improved embryo viability but could not restore morphogenesis, thus permanently affecting embryo development (Figard et al., 2019). This has significant implications for the role of environmental stress in development and illustrates the disruptive impacts of hyperactive cofilin and rod formation beyond neurodegenerative disease.

#### Actin-interacting protein 1

Intranuclear rods formed in *Dictyostelium discoideum* were analyzed for their composition and stability (Ishikawa-Ankerhold et al., 2017). In contrast to cytoplasmic rods, which contain primarily actin and cofilin, intranuclear rods contain additional actin-associated proteins. In this study, actin-interaction protein 1 (Aip1), coronin (CorA), filactin (Fia), and AbpB were identified as components of intranuclear rods by immunofluorescence and mass spectrometry. Rod composition was time-dependent: early rods contain primarily actin and cofilin, while maturing rods incorporated Aip1 and AbpB, and late-stage rods also contained CorA. Aip1 was specifically identified as an important control agent in the assembly and shaping of intranuclear rods (Ishikawa-Ankerhold et al., 2017).

### Effects of cofilin-actin rods

#### Rods block intracellular trafficking

Cofilin has been previously shown to play a role in intracellular trafficking at the Golgi network, potentially through LIMK regulation (von Blume et al., 2011). Cofilin rods have also been shown to inhibit the trafficking of mitochondria and early endosomes by disrupting microtubule integrity and restricting movement to the part of the cell containing the rods (Cichon et al., 2012). Rod formation reduced the number of dendritic spines and impaired the synaptic events associated with neurotransmission. *In vivo*, these rods were found to be present in elderly rat brains but absent in young rat brains. These findings suggest that cofilin rods may advance neurodegeneration and aging in the brain by inducing synaptic loss (Cichon et al., 2012). Rods are also associated with extensive vesicle accumulation within dystrophic neurites (Sanchez-Varo et al., 2012). Dystrophic neurites are one of the early red-flag markers for AD and present cytoskeletal abnormalities, including disruption of stability of neuronal actin filaments. In addition to Aβ plaques, the formation of cofilin-actin rods in the early pathogenesis of AD may result in the accumulation of vesicles in neurites that only further dystrophy (Sanchez-Varo et al., 2012).

#### Rods sequester pTau during Alzheimer’s disease

The presence of persistent cofilin-actin rods and phosphorylated microtubule-associated protein tau (pTau) inclusions in brain tissue are correlated with AD-associated neurodegeneration and cognitive decline (Whiteman et al., 2009). However, the relationship between cofilin rods and Tau inclusions remains an area of active inquiry. In a study of pTau/cofilin-actin rod association, cultured primary neurons subjected to mitochondrial inhibition (ATP depletion) formed striated pTau-containing rods resembling those observed post-mortem in the brains of AD sufferers. These striations were also found to contain, to varying degrees, colocalized cofilin, actin, and actin-depolymerizing factor (ADF). Further studies showed that the observed rod formation was dependent on cofilin/ADF phosphorylation (inactivation). Subsequent overexpression of cofilin-GFP in hippocampal neurons revealed that cofilin-GFP rods formed as a result of ATP depletion were able to sequester pTau, whereas siRNA knockdown of ADF/cofilin eliminated pTau recruitment to rods under ATP depleted conditions (Whiteman et al., 2009). Treatment of neurons with Aβ oligomers also resulted in the formation of ADF/cofilin-pTau associated rods, albeit at levels lower that those observed with direct mitochondrial inhibition. Based on these studies, an increase in cofilin-actin rods may initiate or be a precursor to neuropil threads, leading to synaptic loss and progression of AD.

#### Rods recruit microtubule binding domain epitopes

Mitochondrial function is known to be altered in AD, but the direct link between AD and mitochondrial dysfunction remains an active area of inquiry (Whiteman et al., 2011). Hyperphosphorylated tau aggregates into neuropil threads and contributes to AD pathology. Whiteman et al screened a series of antibodies to track and measure the movement of phosphoepitope tags on tau during mitochondrial inhibition. Results showed epitopes flagged by one of the antibodies, 12E8 (which recognizes residues S262/S356 in humans, or ‘KXGS’ motifs, in microtubule binding domains of tau), were recruited into cofilin-actin rods (Whiteman et al., 2011). Tau hyperphosphorylation could influence cofilin-actin rod formation and affect neuronal activity during periods of mitochondrial distress.

#### Rods concur with Tau pathology but lack colocalization *in vivo*

The correlation between Tau pathology and cofilin-actin rods were investigated in post-mortem brain samples taken from AD patients (Rahman et al., 2014). Abundant phospho-Tau and cofilin rod pathologies were observed in samples from AD patients, but not in young or normal aging control patients. Both density and sizes of cofilin-actin rods observed in hippocampus and the inferior temporal cortex were greater than those observed in non-diseased brain tissue; the observed correlation with tau pathology occurred independently of subject age. In co-localization studies, cofilin-actin rods had minimal overlap with phospho-Tau (Rahman et al., 2014). Although this result contrasts to AD models showing cofilin-actin rods as initiators of Tau pathology (Whiteman et al., 2009), such early events may not be captured by post-mortem analysis of advanced disease. Furthermore, other mechanisms involving activated cofilin and Tau could be at play. In a recent report, activated cofilin was shown to disrupt Tau binding to microtubules (Woo et al., 2019). This cofilin-dependent displacement of Tau also promoted Tauopathy *in vivo* and represents another important link between cytoskeletal dysregulation and disease progress (Woo et al., 2019).

#### Rods part of a feedforward mechanism for Alzheimer’s disease

Rod-like inclusions have previously been found near Aβ plaques in the brains of humans with AD. These rods were also present in the brains of transgenic AD mice and that soluble Aβ plaques induced the rods, dependent on both time and concentration and by way of cofilin dephosphorylation (Maloney, 2005). Also accumulating at these rods were β-secretase-cleaved APP and vesicles containing APP, β-amyloid cleavage enzyme, and presenilin-1. The formation of these rods could prevent the transport of APP and enzyme-containing vesicles to Aβ for processing and could further produce excess Aβ, leading to a feedback loop of rod formation in adjacent neurons and an expansion of the region of neurodegeneration (Maloney, 2005).

## Genetically encoded reporters of cofilin-actin rod formation and dynamics

**FIGURE 4:**
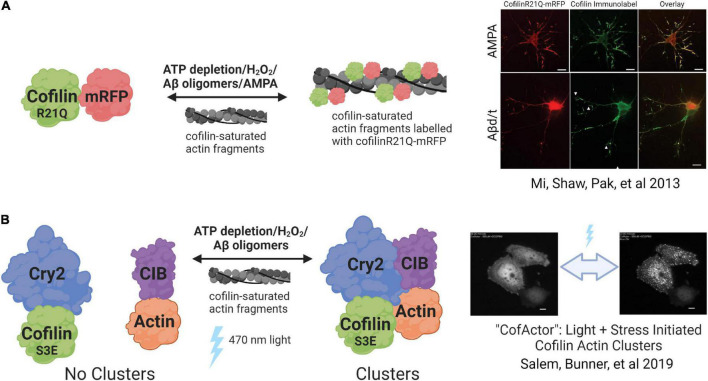
Genetically encoded cofilin-actin rod reporters. **(A)** CofilinR21Q-mRFP incorporates into cofilin-actin rods formed under various stress-inducing stimuli. The association is reversible once the stress solution is removed from cells. **(B)** CofActor (Cry2-Cof.S3E and Actin-CIB) forms rod-like structures in response to a combination of stress-inducing stimulus and blue light. The clusters revert to the non-associated state once the blue light is turned off. Created with www.biorender.com.

In this section, we focus on recent methods for studying cofilin-actin rods in living cells, highlighting their applications in unraveling of the role of cofilin-actin rods in neurodegenerative disease (Figure 4). Currently available options for studying cofilin-actin rod formation and associated phenomena in living cells are rather limited, and thus these reports represent an emerging field. While cofilin-actin rod capture has been achieved through careful and well-documented methods for their fixation, immunostaining, and isolation (Minamide et al., 2010; Won et al., 2018), these new methods offer the advantage of monitoring rod formation in real-time. Capturing rod formation and its downstream impacts in this dynamic context will more precisely define the impacts of cofilin-actin rods and will facilitate next-generation therapeutic strategies by enabling the kinetic profiling of rod formation and elimination.

### A genetically encoded reporter

A major challenge encountered when using cofilin-based fluorescent probes is their tendency to spontaneously form cofilin-actin rods under conditions of homeostasis (Jang et al., 2005; Bernstein et al., 2006; Cichon et al., 2012). As a result, any strategy for monitoring cofilin-actin rods in living cells that involves WT cofilin overexpression must be carefully designed to avoid this complication. Mi et al. (2013) developed a cofilin mutant (R21Q) that does not readily incorporate into rods when expressed in neurons (Figure 4A). The R21Q mutant was selected from a pool of 13 point mutations to cofilin residues predicted to interact directly with the F-actin interface (K13Q, R21Q, K22Q, S23A, S24A, T25A, T25G, E27A, E27G, K31R, T32L, K95Q, C39A, C139A, AND C147A) expressed as fusions to mRFP (Mi et al., 2013). These mutants were assessed for their resistance to spontaneous formation, and the R21Q mutant emerged as having the most desirable properties (low background rod incorporation; high rod incorporation under ATP-depleted conditions) in LLC-PK_*A*4_._8_ cells. This mutant was subsequently shown to enable monitoring of rod dynamics and was reversible when sources of applied cellular stress (AMPA, ATP depletion, or Aβ) were removed (Mi et al., 2013). The cofilinR21Q-RFP probe was then used to monitor the impacts of rod formation on synaptic vesicle trafficking in AMPA-treated neurons. Overall, CofilinR21Q-mRFP represents a first-in-class, genetically encoded reporter of cofilin-actin rod formation that will further define the impact of rods in synaptic function (Mi et al., 2013).

### CofActor: A light and stress-gated optogenetic clustering tool

Another genetically encoded reporter of cofilin-actin rod dynamics is then light activated CofActor (Cofilin Actin optically responsive) system (Figure 4B) (Salem et al., 2020). This optogenetic cassette, based on the blue light activated Cryptochrome2/CIB protein pair, consists of two protein fusions: Cry2-mCherry-Cof.S3E and βActin-CIBN-GFP. Similar to the mutagenesis strategy employed in the generation of CofilinR21Q above, the Cofilin S3E mutation prevents cofilin from spontaneously incorporating into cofilin-actin rods. In addition, this mutation, which mimics the biological “off” state of cofilin when phosphorylated at Ser3, resists interaction with actin even under the energetic and oxidative stress conditions required for sustained cofilin-actin rod formation. As a result, the CofActor system is a dual-input biosensor that requires both light *and* oxidative or energetic stress to report on cofilin-actin rod formation. In this initial report, the CofActor switch response was demonstrated in both HeLa cells (Salem et al., 2020) and in primary hippocampal neuron cultures (Salem et al., 2020; Bunner et al., 2021). Furthermore, mutagenesis studies revealed that cysteine residues 39 and 147 in cofilin were critical for CofActor function, as was actin residue S14, which forms a hydrogen bond with the gamma phosphate of ATP. Mutation of S14 to Alanine or Valine resulted in a CofActor variant with a stress-independent rod-forming response (Salem et al., 2020), suggesting that it may be possible to engineer a cofilin-actin rod forming switch that will enable spatial and temporal control of rod formation under conditions of homeostasis. Methods for incorporating the CofActor system into neurons have been recently reported (Bunner et al., 2021), and CofActor plasmids are available through Addgene. The authors report that additional development of this optogenetic system is currently underway.

## Small molecule, peptide, and protein mediators and inhibitors of cofilin-actin rods

Cofilin-actin rods remain an intriguing therapeutic target in neurodegenerative disease. Strategies for controlling cofilin-actin rods range from direct targeting of cofilin and actin to modulation of the various cell signaling pathways upstream of rod formation (Shaw and Bamburg, 2017). While a general solution for rod regulation remains elusive, the general lack of effective therapies for Alzheimer’s disease and its increasing incidence makes all potential therapeutic targets, including cofilin-actin rods, deserving of further investigation. In this section, we describe strategies for the control of cofilin-actin rod formation, ranging from small molecule to protein-based therapeutics (summarized in Table 1). If these strategies eventually result in clinical vectors, they could be important tools for the management of a host of neurodegenerative diseases.

**TABLE 1:** Proteins, peptides, and small molecule inhibitors of cofilin-actin rods.

Name	Target	Function
**Small molecules**		
Tetracyclines*	Actin	Reduces overall size of the complete actin aggregate (Pathak et al., 2021).
Miuraenamide*	Actin	Binds to actin monomers by manipulating the DNase-1 binding which aids polymerization; prevents cofilin binding (Wang et al., 2021).
Pyrollopyridines^†^	LIMK	Inhibits LIMK through its ATP binding site, inhibiting cofilin phosphorylation (Ariawan et al., 2021).
Deubiquitinase Inhibitors^†^	Deubiquitinase	Promote F-actin assembly independently of other pathways or through cofilin dephosphorylation (Larbret et al., 2022).
Cucurbitacin-B*	VASP	Inactivates VASP by phosphorylation and prevents actin remodeling, resulting in actin aggregation (Zhang et al., 2014).
Pyr1^†^	LIMK	Inhibits LIMK, leading to increased cofilin activation and actin dynamics (Gory-Fauré et al., 2021).
NOX Inhibitors*	NOX	Various (Vas2870, et al) inhibitors of NOX prevent ROS production (Barua et al., 2019).
**Peptides**		
Cofilin-derived peptides*	Cofilin, LIMK, or SSH1	Regulates different targets in the actin-cofilin pathway depending on the design of the peptide (Shaw and Bamburg, 2017).
**Proteins**		
SGK1^†^	Ion channels, Na^+^/K^+^-ATPase	Up-regulates the activity and expression of many ion channels and Na^+^/K^+^-ATPase (Lang et al., 2010).
Hsp70^†^	Unfolded or misfolded proteins	Assists in folding of new proteins, proofreading and refolding of misfolded proteins, and translocation of proteins to organelles (Mayer and Bukau, 2005).
LIMK*	Cofilin	Phosphorylates and inactivates cofilin (Zhang et al., 2021a).

*A direct link has been found to cofilin-actin rods.

^†^Hypothesized link to cofilin-actin rods.

### Small molecule mediators and inhibitors

#### Tetracycline family

The aggregation of actin into Hirano bodies is a trademark of many neurodegenerative diseases (Mitake et al., 1997). In *Saccharomyces cerevisiae*, these aggregates were treated with tetracycline, oxytetracycline, doxycycline, and minocycline to investigate their effects on the size of Hirano bodies (Pathak et al., 2021). Treatment with the tetracycline molecules bound to and reduced the size of overall actin aggregates at varying concentrations, depending on the speed and efficiency recorded by each of the various molecules. Tetracycline and oxytetracycline were found to be the most effective in the family based on efficiency of aggregate destruction, size of particulates after treatment, and extent of disaggregation compared to minocycline and doxycycline, which were less effective at comparable concentrations. These antibiotics were shown to be a potential treatment for early Alzheimer’s patients by destroying actin aggregates prior to Hirano body formation and disrupting the progression into neurodegenerative disease (Pathak et al., 2021).

#### Pyr1 and pyrrolopyrimidine-based LIM kinase inhibition

The correlation between LIMK and actin dynamics can be investigated through LIMK inhibitors, such as Pyr1 (Gory-Fauré et al., 2021) and pyrrolopyrimidine-based inhibitors (Ariawan et al., 2021). Both Pyr1 and pyrrolopyrimidine compounds have been found to be highly selective and capable of stabilizing microtubules and modulating actin dynamics (Ariawan et al., 2021; Gory-Fauré et al., 2021). Long-term treatment of mice with Pyr1 resulted in an overall decrease of inactive cofilin, indicating an inhibition of LIMK (Gory-Fauré et al., 2021). Similarly, derivatives of the pyrrolopyrimidine scaffold were also effective at inhibiting the activity of LIMK1, resulting in inactive cofilin (Ariawan et al., 2021). LIMK inhibition results in increased active cofilin and cofilin-actin rod formation and could be a useful method for studying the effects of aberrant cytoskeletal dynamics.

#### Deubiquitinase inhibition

Multiple deubiquitinase (DUB) inhibitors were investigated for F-actin assembly modulated by cofilin using flow cytometry and FRET (Larbret et al., 2022). Through the treatment of leukemic T cells with inhibitors WP1130 and b-AP15, DUB inhibition was found to cause a dramatic redistribution of actin to the cell membrane, preventing cell migration (Larbret et al., 2022). By inducing DUB inhibition, actin reorganization involved a non-degradative and LIMK-independent modulation of cofilin activity, accumulation of polyubiquitinated proteins, and generation of ROS (Larbret et al., 2022). This response resulted in the oxidation and dephosphorylation of cofilin and may present another means of studying conditions leading to cofilin-actin rod formation.

#### Cucurbitacin-B

Cucurbitacins are a family of small molecules that disrupt the actin cytoskeleton (Zhang et al., 2013), leading to cofilin actin rod formation (Zhang et al., 2014) and the detriment of various essential cellular functions, such as cell structure and cell cycle progression (Duncan et al., 1996). Cucurbitacin-B (CuB) is a potent antineoplastic agent and has been extensively studied for its anti-cancer properties (Chen et al., 2012). In a recent study involving its role in actin signaling pathways, CuB treated cells showed an increase in actin aggregation followed by reorganization into cofilin-actin rods (Zhang et al., 2014). Gα13 and RhoA were found to be upstream regulators of CuB-induced VASP phosphorylation at Ser 157, as exhibited by the significant suppression or full blockage of actin aggregation and cofilin dephosphorylation upon knockdown of Gα13 or RhoA gene expression (Zhang et al., 2014). Ultimately, VASP phosphorylation through Gα13/PKA/RhoA signaling pathways played a crucial role in CuB-induced actin aggregation and cofilin-actin rod formation. Although the mechanism by which Gα13 is regulated by CuB has yet to be fully elucidated, investigation into CuB-induced cofilin-actin rods provides an area of therapeutic interest.

#### Fasudil

Increased ROCK levels and elevated phosphorylated cofilin have been observed in AD brains (Henderson et al., 2016). Fasudil, a ROCK inhibitor, prevents impairment and loss of synapses through inhibition of cofilin phosphorylation (Rush et al., 2018). Experiments performed by Rush et al. (2018) showed that Aβ plaques stimulated ROCK activity and increased phosphorylated cofilin with subsequent F-actin stabilization in the dendritic spines of primary cortical neurons. The addition of Fasudil inhibited the Aβ-induced cofilin phosphorylation, which rescued synaptic loss associated with Aβ (Rush et al., 2018). While the inhibition of ROCK may seem non-intuitive considering its role in LIMK activation, increased ROCK activity induced by Aβ can stimulate β-secretase and γ-secretase activity, which in turn hydrolyzes APP to Aβ. Inhibition of overactive ROCK rescues synaptic damage caused by Aβ and prevents further APP cleavage (Cai et al., 2021). Interestingly, this model of cofilin *inactivation* as a driver of AD pathology is seemingly in conflict with the cofilin-actin rod hypothesis: elevated levels of active cofilin are proposed to drive the disease state, indicative of the key, yet unresolved role of cofilin in neurodegenerative disease.

#### NADPH oxidase inhibitors

NOX activation results in the production of ROS, a known promoter of cofilin-actin rods. In models of AD, PD, and ischemic stroke, NOX inhibition has resulted in neurological improvement associated with a cognitive deficit reversal and neuroprotective properties (Barua et al., 2019). In the context of neurodegenerative disease, many direct inhibitors of NOX have been identified: NOX2 docking sequence-tat (Nox2ds-tat), diphenyleneiodonium (DPI), Honokiol, Plumbagin, Ebselen, phenylarsine oxide (PAO); and indirect inhibitors: Vas2870, apocynin, and statins (Barua et al., 2019). While not an exhaustive list, NOX inhibitors have become attractive as a blocker of Aβ-induced ROS generation via NOX. Inhibitors such as Noxds-tat and Eblesen prevent the NOX cytosolic subunit p47^phox^ from interacting with its membrane-bound subunit p22^phox^, inhibiting the activation of NOX (Barua et al., 2019). Vas2870 specifically is a readily available and widely used NOX inhibitor which indirectly prevents NOX stimulation. In cell-based experiments, Vas2870 has been proven to be a potent inhibitor of cofilin-actin rods (Tam et al., 2019; Salem et al., 2020).

### Peptide regulation of rod formation

#### Miuraenamide A

The myxobacterial depsipeptide miuraenamide A, has been shown to disassemble F-actin cytoskeletal networks at relatively low concentrations (Wang et al., 2021). Initially, miuraenamide A was computationally determined to bind strongly to tightly-packed actin monomers by manipulating the actin DNase-1 binding loop, a key factor in F-actin stabilization (Wang et al., 2019). As a result, miuraenamide A was hypothesized to be capable of disrupting actin polymerization and be implicated in cofilin-actin rod inhibition. From a pool of related derivatives, miuraenamide A was the only compound to interact with F-actin at a concentration lower than 100 nM, while also inducing smaller aggregates of actin monomers throughout the cytoplasm, compared to actin aggregation of the derivatives at the cell membrane (Wang et al., 2021). Miuraenamide A can compete with phalloidin’s binding to actin, suggesting that miuraenamide A may share the same binding site as phalloidin (Wang et al., 2019). Furthermore, when miuraenamide A interacts with actin, the DNase-1 binding loop rearranges and its cofilin binding site is obstructed by tighter packing of actin monomers (Wang et al., 2019). As a potential competitor for cofilin binding, Miuraenamide A may be used to prevent the formation of cofilin-actin rods.

#### Peptides derived from actin binding proteins

Cofilin-derived peptides have also been utilized for regulation of cofilin activity (Shaw and Bamburg, 2017). The S3 peptide (cofilin residues 1-16: MASGVAVSDGVIKVFN) inhibits LIMK and promotes cofilin activity (Aizawa et al., 2001), while the pS3 peptide [cofilin 1–16 with phosphoSer3: MAS(p)GVAVSDGVIKVFN] inhibits SSH1 and subsequently inhibits cofilin activity (Zhou et al., 2004). Aizawa et al. (2001) coupled the S3 peptide with penetratin, a cell penetrating peptide, enabling uptake directly from the cell culture medium. To rescue synaptic function in a Shank3 KO mice model of ASD, the pS3 peptide was fused to the HIV protein TAT for cellular uptake and injected intravenously into the mice (Duffney et al., 2015). F-actin assembly increased and cofilin activity decreased after TAT-pS3 peptide injection (15 pmol/g), while also improving behavioral abnormalities in the mice. In the same study, an injection of a peptide derived from 18 amino acids of the proline-rich domain of PAK1 (TAT-PAK18) competed with endogenous PAK and reduced PAK (Duffney et al., 2015). Other peptides derived from actin binding proteins have been developed for their therapeutic effects in brain injuries, such as AcSDKP peptide from protein thymosin β4 (Zhang et al., 2016), demonstrating that mining actin binding proteins for therapeutic peptides can be a general strategy for inhibiting actin-dependent phenomena such as cofilin-actin rods.

### Protein regulation of rod formation

#### Hippocampal overexpression of serum- and glucocorticoid-inducible kinase 1

Serum- and glucocorticoid-inducible kinase 1 is a member of the Ser/Thr protein kinase family and is involved in spatial memory formation and consolidation in the hippocampus (Lian et al., 2020). A recent study revealed a lack of SGK1 in AD model mice; overexpression of SGK1 in the hippocampus of these mice directly resulted in improved memory (Lian et al., 2020). SGK1 overexpression increased the number of neurites and shortened the time required for neurite formation in hippocampal neurons, in addition to a reduction of Aβ plaques as a result of an increase in Aβ degradase expression (Lian et al., 2020). As AD model mice lack SGK1, the resultant under expression of Aβ degradase could attenuate accumulation of Aβ oligomers in neurons, inducing cofilin-actin rod formation. Further studies are necessary to determine if SGK1 overexpression could be utilized to reduce Aβ-induced cofilin-actin rod formation in AD model mice.

#### 70 kDa inducible heat shock protein

The 70 kDa inducible Hsp70 is a chaperone that facilitates protein folding, assembly, and degradation; Hsp70 has been previously proposed as a method for reduction of neurological impairment and lesions following ischemic injury (Kim et al., 2018). In a recent study, the neurological deficits of overexpressed (Tg) and knockout (KO) Hsp70 mice were compared before and after an occlusion simulating a stroke (Kurisu et al., 2019). Compared to wild type mice, Hsp70KO showed a significant increase in cofilin-actin rod formation at both 4 and 24-h post occlusion, whereas Hsp70Tg showed decreased cofilin-actin rod expression. Thus, therapeutic overexpression of Hsp70 presents a novel means of reducing cofilin-actin rod formation and a promising therapeutic for damage reduction and neuroprotection post-stroke (Kurisu et al., 2019).

#### LIM kinase

*In vivo*, dephosphorylated cofilin has been shown to translocate to mitochondria and assist in apoptosis (Madineni et al., 2016). In APP/PS1 mice model of AD, LIMK1 expression increased phosphorylation, therefore decreasing the activity of cofilin in hippocampal neurons (Zhang et al., 2021a). LIMK1 activity also recovers LTP impairments with subsequent improvements in memory formation (Zhang et al., 2021a). In a separate study, overexpression of LIMK via lentivirus infection in mice brains decreased cofilin-actin rod density and reduced cell apoptosis in the area of injection (Chen et al., 2020). The extrinsic activation of LIMK could provide a therapy for individuals post-ischemic stroke by repairing and preventing the degenerative effects of cofilin-actin rods and apoptosis.

## Other actin-associated proteins involved in cytoskeletal dysregulation and neurodegenerative disease

Numerous actin-associated entities, ranging from actin-associated scaffolding proteins and enzymes to receptor targeting peptides, play important roles in disease-related cytoskeletal dysregulation. A comprehensive overview of these entities might facilitate identification of therapeutic nodes in neurodegenerative disease. Furthermore, this analysis could further in the development of next generation strategies for the treatment of AD and associated disorders. In this section, we describe some of the familiar entities (Rho, ROCK, etc.) that are commonly regarded as master regulators of the actin cytoskeleton, while also highlighting additional proteins (summarized in Table 2) that are emerging as important cytoskeletal regulators in neurodegenerative disease and likely play a role in cofilin-actin rod dynamics.

**TABLE 2:** Actin-binding and actin-associated proteins involved in cofilin-actin rod regulation.

Name	Target	Function
Arp2/3 complex^†^	Actin	Facilitates actin branching in dendritic spines (Zhang et al., 2021b).
ATM kinase^†^	Drebrin	Phosphorylates drebrin under ROS stress (Kreis et al., 2019).
Bin1*	Actin	Binds and stabilizes F-actin and tau actin bundles; induces actin bundling (Dräger et al., 2017).
CAP^†^	Actin, cofilin	Regulates actin treadmilling for synaptic plasticity (Di Maio et al., 2022).
Cdc42*	PAK1, WASP and Arp2/3 complex	Activates PAK1 leading to actin filament assembly; activates Arp2/3 complex through WASP signaling leading to actin branching (Zhang et al., 2021b).
Cellular prion protein (PrPc)*	NOX	Activates NOX pathway and leads to ROS generation (Walsh et al., 2014a).
Cdk5^†^	Drebrin	Activates drebrin by phosphorylation (Gordon-Weeks, 2016).
Drebrin*	Actin	Stabilizes actin filaments and regulates synapse structure associated with cognitive ability (Liu et al., 2017).
EB3^†^	Drebrin, microtubules	Connects actin filaments to plus-end of microtubules (Gordon-Weeks, 2016; Yamazaki and Shirao, 2017).
Egr-1^†^	Drebrin	Binds to the drebrin promoter and prevents drebrin expression (Cho et al., 2017).
Fe65^†^	APP, Mena	Scaffolding protein that interacts with APP; indirectly induces actin polymerization through Mena (Augustin and Kins, 2021).
Homer^†^	Shank, drebrin	Recruits PSD-95 and active Cdc42 to F-actin to produce dendritic spines (Tu et al., 1999).
Huntingtin*	Nuclear rods	Localizes to nuclear actin-cofilin rods under stress and facilitates actin dynamics (Munsie et al., 2011).
PAK1*	LIMK, SSH1	Activates LIMK and inactivates SSH1; regulates cofilin, leading to actin filament assembly (Zhang et al., 2021b).
Pin1*	pSer/Thr-pro motifs	Isomerizes proteins containing pSer/Thr-Pro motifs; associated with Tau and APP (Balastik et al., 2007; Wu et al., 2017)
Profilin*	Actin	Inhibits actin polymerization (Walter et al., 2021).
Progranulin^†^	Drebrin	Assists in dendritic spine formation (Xu et al., 2015).
PRRT2*	SNARE complex, cofilin	Facilitates neurotransmitter release through interaction with SNARE complex; modulates cofilin’s function in actin dynamics (Savino et al., 2020).
PTEN^†^	Drebrin	Inactivates drebrin by dephosphorylation (Yamazaki and Shirao, 2017).
Rac1*	PAK1, WAVE and Arp2/3 complex	Activates PAK1 and Arp2/3 complex through WAVE signaling (Zhang et al., 2021b).
RanBP9*	SSH1, Aβ	Scaffolding protein that upregulates SSH1, leading to cofilin activation; increases Aβ protein (Woo et al., 2015).
RhoA*	ROCK	Activates ROCK, leading to actin assembly (Zhang et al., 2021b).
ROCK*	LIMK, SSH1	Activates LIMK and inactivates SSH1 leading to actin filament assembly (Zhang et al., 2021b); potential role in downregulation of autophagy (Weber and Herskowitz, 2021).
Spikar^†^	Drebrin	Stabilize the formation of dendritic spines (Yamazaki et al., 2014).
SSH1*	Cofilin, LIMK	Induces actin polymerization by activating cofilin via dephosphorylation; inhibits LIMK (Soosairajah et al., 2005).

*A direct link has been found to cofilin-actin rods.

^†^Hypothesized link to cofilin-actin rods.

### Drebrin/EB3

The Drebrin/EB3/Cdk5 signaling pathway connects actin filaments to microtubules during neuronal development and during dynamic membrane remodeling along dendritic spines (Gordon-Weeks, 2016). Developmentally regulated brain protein *Drebrin* specifically binds and stabilizes actin filaments for regulation of synapse structure and dynamics in dendrites associated with long-term memory (Gordon-Weeks, 2016). Activation of Cyclin-dependent kinase 5 (*Cdk5*) phosphorylates Drebrin, which undergoes an “open” conformational change allowing drebrin to bundle other actin filaments. *End-binding* (*EB3*) *protein* performs plus-end tracking (+TIP) on an elongating microtubule, for example in the filopodium of a growth cone, and can interact with the “open” Drebrin on actin filaments, connecting the filaments to the microtubule. Since this pathway is active during spine maturation, it can regulate synaptic plasticity and memory formation (Gordon-Weeks, 2016). As Drebrin regulates F-actin dynamics and stability in dendritic spines, a loss of Drebrin in spines is associated with synaptic loss and cognitive decline in AD models (Liu et al., 2017). Without Drebrin, dendritic spines would be constantly dynamic due to the unstable F-actin; therefore, LTP could not occur. Correspondingly, AD model mice display atypical LTP (Shirao et al., 2017). *Drebrin and cofilin:* There is an inverse correlation between Drebrin levels and cofilin activity: as active cofilin levels increase, Drebrin levels decrease (Zhao et al., 2006). Drebrin’s actin-binding site shares sequence homology with cofilin’s actin-binding site and activated cofilin competes with Drebrin for actin binding in dendritic spines (Zhao et al., 2006). Interestingly, the LIMK activator, PAK, is under-expressed in AD brains, resulting in increased cofilin activity and loss of Drebrin in actin filaments due to competitive binding. In AD models, the presence of Aβ plaques directly contributed to PAK dysfunction, Drebrin loss, and cognitive deficits (Zhao et al., 2006). *In vivo* increase in Drebrin levels could competitively remove cofilin from cofilin-actin rods and thereby rescue synaptic activity (Liu et al., 2017). Additionally, the restoration of PAK expression and function could be another target for cofilin-actin rod prevention. *Drebrin in other conditions:* Drebrin is not only reduced in AD-afflicted brains, but also in other neurological conditions including amyotrophic lateral sclerosis (ALS), Down Syndrome, bipolar disorder, mild cognitive impairment (MCI), and SCZ. Drebrin naturally decreases during the aging process and has been linked indirectly to familial frontotemporal dementia (FTD) (Shirao et al., 2017). As Drebrin is an integral component of neuronal function and cognitive ability, identifying therapies for restoring Drebrin expression could be beneficial.

### Drebrin-related proteins

Drebrin expression is down-regulated by the transcription factor, *Early growth response-1* (*Egr-1*), which binds to the Drebrin promoter and prevents Drebrin expression. In corroboration, overexpression of Egr-1 in hippocampal neurons resulted in a decrease in Drebrin, in addition to reduction of dendritic spines and expression of synaptic markers. The opposite effects occurred in Egr-1 knockdown mice where Drebrin expression increased (Cho et al., 2017). Inhibitors of Egr-1, such as the previously developed phosphorothioate antisense oligonucleotides (Baron et al., 2003), could be utilized to up-regulate Drebrin in AD patients. *Homer* is localized in the cytoplasm of dendritic spines and plays a role in the signaling pathway that modulates spine morphogenesis (Yamazaki and Shirao, 2017). Homer interacts with the NMDAR-associated postsynaptic family of Shank proteins to recruit additional proteins, such as F-actin, postsynaptic density (PSD)-95, and active Cdc42, and subsequently increase dendritic spine length (Tu et al., 1999). Homer may also interact with Drebrin located on F-actin filaments for localization of Homer complexes to dendritic spines. Drebrin may also interact with a Homer-Cdc42 complex for spine formation (Yamazaki and Shirao, 2017). *Spikar*, another Drebrin-associated protein, assists in the stabilization and formation of dendritic spines. Nuclear Spikar is a DNA damage response factor that aids chromatin repair mechanisms; however, in neurons, Spikar is localized to the cytoplasm of dendritic spines where Drebrin is accumulated. Drebrin binds Spikar to dendritic spines, increasing spine density without altering morphology (Yamazaki et al., 2014). *Profilin* and Drebrin exhibit affinity to each other *in vitro* and can separately accumulate in dendritic spines after LTP, suggesting a profilin-drebrin interaction induced by LTP (Mammoto et al., 1998); however, additional studies on how profilin affects Drebrin function are necessary. *Progranulin* plays many important physiological roles in cytoskeletal remodeling such as cell proliferation and inflammation, and mutations of its gene are implicated in FTD, AD, and cancer (Yamazaki and Shirao, 2017). Progranulin induces actin dynamics crucial for cell migration, and in cancer cells, Drebrin was found to interact with progranulin during this process (Xu et al., 2015). Similar to other proteins mentioned, knockdown of progranulin results in a reduction of dendritic spines and an increase in smaller protrusions resembling filopodia (Tapia et al., 2011). The similar function, localization, and result of knockdown studies indicate a correlation between progranulin and Drebrin and its related signaling pathways associated with spine formation. *Phosphatase and tensin homolog* (*PTEN*), a tumor suppressor, dephosphorylates Drebrin and has been implicated in AD. PTEN is recruited to dendritic spines by Aβ induction; however, in AD models, this recruitment is reduced. PTEN modulates NMDAR activation in long-term depression (LTD); over-activity of NMDAR induces Drebrin degradation (Yamazaki and Shirao, 2017). Drebrin regulates cytoskeletal dynamics assisted by *ataxia-telangiectasia mutated* (*ATM*) *kinase* (Kreis et al., 2019). A rare genetic mutation in the ATM gene causes its namesake neurodegenerative disease, ataxia-telangiectasia. Under stress conditions such as ROS, Drebrin is phosphorylated by ATM which leads to the stabilization of dendritic spines. If Drebrin is constantly phosphorylated, as in a p-Drebrin mutant, ATM can no longer stimulate this phosphorylation response after oxidative stress and cannot protect the neuron from synaptic dysfunction. ATM phosphorylation of Drebrin allows for cytoskeletal remodeling and increases the cell’s resistance to stress. In AD, ATM-regulated Drebrin protects against synapse degeneration due to actin loss and subsequent cognitive decline (Kreis et al., 2019).

### Rho family small GTPases

Rho family small GTPases assist in downstream signaling for the regulation of actin dynamics (Zhang et al., 2021b). Rho proteins regulate synaptic transmission, LTP, and LTD. *Cdc42* is crucial for inducing LTP, whereas *RhoA* assists in maintaining LTP. *Rac1* helps destabilize LTP and induce LTD. The Rho GTPase effectors, PAK and ROCK, are also regulators of synaptic plasticity and mediate LTP and LTD. The downstream target of the effectors, LIMK, inhibits actin depolymerization, and its absence results in shortened dendritic spines and diminished LTP ability. SSH1 can be inactivated by PAK and ROCK; therefore, cofilin activation can then be indirectly regulated by the effectors (Zhang et al., 2021b). Rac1 and Cdc42 additionally regulate the actin nucleation factor, *Arp2/3 complex*, which facilitates actin branching during dendritic spine remodeling. RhoA and Cdc42 are both implicated in memory formation, whereas Rac1 has a role in memory elimination but can be regulated to escalate or hinder memory decay (Zhang et al., 2021b).

Rho GTPases play a role in AD pathology. In the neurons of AD models, Aβ peptides induce aberrant actin-cofilin rods in dendritic spines that lead to synapse loss and cognitive deficits. In APP/PS1 mice and post-mortem human brains of AD patients, Rac1 activity was upregulated, most likely induced by Aβ peptides (Wu et al., 2019), and other studies have further suggested that heightened Rac1 activation correlates with rapid memory loss and is age-dependent (Zhang et al., 2021b). Aβ can induce ROS in a Rac1 dependent manner, and Rac1 can also increase Aβ formation, indicating a bidirectional feedback loop that exacerbates AD symptoms (Gianni et al., 2003).

Cdc42 is also upregulated in AD brains through Aβ induction and increases actin filament formation and elongation. RhoA was found to have the opposite effect: a decrease of activity in AD brains; however, RhoA is similarly involved in Aβ peptide production, potentially in cooperation with Rac1 and Cdc42 (Mendoza-Naranjo et al., 2007). An overproduction of Aβ can induce cofilin-actin rods, therefore regulation of Rho family small GTPases may prevent rod formation.

### Rho-associated coiled-coil protein kinase

ROCK2 is a serine/threonine kinase that is activated downstream of RhoA and regulates actin dynamics via LIMK and cofilin (Weber and Herskowitz, 2021). Studies in neurons have suggested ROCK2 regulates autophagy (Gurkar et al., 2013; Koch et al., 2014). Misfolded tau aggregates are normally degraded and removed from the cell via autophagy to prevent toxicity and cell death; however, in AD, the presence of these toxic tau oligomers could be caused by autophagy dysregulation (Weber and Herskowitz, 2021). Inhibition of ROCK2 can induce autophagy and therefore decrease Aβ in the cell. Furthermore, ROCK2 inhibition can encourage actin cytoskeleton rearrangement, thus increasing Aβ resilience in neurons and reducing incidence of rod formation. Fasudil or the inhibitor SR3677 might be a promising therapeutic for AD as it can inhibit ROCK2 and decrease Aβ production. ROCK2 may interact with mTORC pathways regulating autophagy, and the inhibition may also promote autophagy of tau and Aβ aggregates (Weber and Herskowitz, 2021). Prevention of tau aggregate formation through ROCK inhibition might alleviate AD symptoms by increasing actin cytoskeleton dynamics, subsequently rescuing synapse loss.

### Cyclase-associated proteins

Another actin-binding protein, CAP, regulates actin treadmilling of F-actin in hippocampal neurons (Schneider et al., 2021; Di Maio et al., 2022). CAP1 is integral to the function of growth cones along with neuron differentiation and connectivity, whereas CAP2 modulates synaptic plasticity and spine morphology in mature neurons. Using knockout experiments, cofilin1 and CAP1 were found to mutually rely on each other for correct function in growth cones (Schneider et al., 2021). CAPs are dendritic spine regulators – these proteins assist in actin remodeling at the postsynaptic density (PSD) of dendritic spines and could play a role in abnormalities in SCZ and neurodegenerative diseases. A role for CAP2 in cofilin regulation in AD has recently been proposed (Pelucchi et al., 2020). CAPs are implicated in both actin and cofilin regulation, thus alteration of these proteins may play a role in the formation of cofilin-actin rods.

### RanBP9

One promising therapeutic method for Alzheimer’s disease includes alteration of the production and downstream pathways of Aβ plaques in Alzheimer’s patients. Overexpression of scaffolding protein RanBP9 promotes Aβ production, induction of mitochondrial stress, and upregulation of SSH1 expression levels with subsequent cofilin activation (Woo et al., 2015). In fAPP/PS1 mice exhibited significant increases in RanBP9 expression. In studies of RanBP9 knockdown (*RanBP9*^±^) in APP/PS1 mice, RanBP9 was a necessary mediator of cofilin translocation to the mitochondria in response to Aβ oligomer treatment. To corroborate RanBP9’s indirect role on cofilin activation, a fewer percentage of *RanBP9*^±^ neurons exhibited cofilin-actin rods when under Aβ oligomer induction than wildtype APP/PS1 neurons. Knockdown of RanBP9 also prevented depletion of Drebrin, PSD95, and F-actin within dendritic spines in addition to Aβ oligomer aggregation, neuroinflammation, and synaptic damage. As a result, this reduction in RanBP9 enhanced LTP and protected against memory deficits exhibited in wildtype APP/PS1 mice (Woo et al., 2015). These results show that RanBP9 is required for cofilin-actin pathology and the hallmark accumulation of Aβ, and thus its reduction facilitates a decrease in the neurotoxicity associated with AD (Woo et al., 2015).

### Fe65 scaffold proteins

Fe65, a scaffold protein family, interacts with and alters the processing of APP (Augustin and Kins, 2021). Fe65 is mainly enriched in the brain, and its overexpression has been shown to reduce Aβ levels in AD model mice (McLoughlin and Miller, 2008). A major group of proteins that interact with Fe65 play a role in actin regulation, including Mena. The interaction between Mena and Fe65 sparked the hypothesis that Fe65 is directly involved in regulation of the actin cytoskeleton. Further investigation has indicated that this interaction specifically occurs when Fe65 attaches to surface proteins that bind to Mena, promoting actin polymerization (Augustin and Kins, 2021). Various protein complexes that interact with Fe65, including ELMO1/DOCK1/Arf6/Rac, indicate that Fe65 could play a role in cofilin-actin rod regulation (Augustin and Kins, 2021).

### Bin1

Bin1, a N-BAR protein, was established as a marker for Alzheimer’s disease due to its ability to shape membranes and its consistent presence in AD patients. Bin1 plays a direct role in actin binding and stabilization, specifically of tau-induced actin bundles. This involvement in actin bundling that has a direct link to AD provides evidence of the role of Bin1 in the development of the disease. A study in a *Drosophila* model of AD showed that downregulation of Bin1 resulted in the reduction of actin inclusions (Dräger et al., 2017).

### Pin1

Peptidyl-prolyl *cis-trans* isomerase NIMA-interacting 1 (Pin1) specifically isomerizes motifs containing a phosphorylated serine or threonine residue directly before a proline residue (pSer/Thr-Pro) (Balastik et al., 2007). This unique enzyme is correlated with numerous diseases, including neurodegeneration. Oxidative stress has been tied to the over-activation of Pin1 (Wu et al., 2017). In neurons, Pin1 can compete with CKS1 for p27/kip1 binding. Liberated p27/kip1 reduces RhoA-mediated cofilin phosphorylation, resulting in cofilin-actin rod accumulation (Kukalev et al., 2017).

### Proline-rich transmembrane protein 2

Proline-rich transmembrane protein 2 mutants are implicated in a wide array of paroxysmal neurological disorders (Savino et al., 2020). In a recent study, silencing of the PPRT2 gene in hippocampal neurons impacted actin dynamics, resulting in a decrease in the density and maturation of dendritic spines (Savino et al., 2020). These changes were associated with the hyperactivation of cofilin, which also resulted in the formation of cofilin-actin rods within neurites. Expression of a constitutively inactive cofilin S3E mutant in PRRT2 KO neurons partially rescued synapse morphology but did not rescue synaptic transmission (Savino et al., 2020).

### Huntingtin protein

Mutations in the gene encoding the Huntingtin protein are an etiology of HD (Munsie et al., 2011). Under stress, cells release endogenous huntingtin from the ER into the nucleus and localize to nuclear actin-cofilin rods, facilitating actin dynamics; however, mutant huntingtin localization to these rods does not allow for restoration of actin and instead produces a persistent phenotype in rods that resemble cytoplasmic rods in AD pathology (Munsie et al., 2011). ATP depletion has been found in the brains of HD patients, and this stress also causes cofilin rod formation in AD. Reduced stress response due to the huntingtin mutant could worsen ATP depletion and further contribute to persistent rods, leading to unavailable cofilin, aberrant actin dynamics, and neuron degeneration (Munsie et al., 2011). Actin-cofilin rods with attached huntingtin mutant have been found to correlate with HD progression in lymphocytes of HD patients. Covalent cross-linking of actin and cofilin had also been observed and is mediated by increased transglutaminase 2 activity, resulting in decreased spine density and eventual synaptic loss. The consequence of this is a feedback loop of cellular stress caused by decreased ATP, which further increases cofilin-actin rod formation (Munsie et al., 2011).

### Chronophin

Chronophin (CIN), a haloacid dehalogenase family hydrolase, can dephosphorylate cofilin during ATP depletion, promoting cofilin-actin rod formation (Huang et al., 2008). CIN is unrelated to SSH1, and its phosphatase activity is increased under reduced ATP conditions. In fact, CIN-activated cofilin resulted in the formation of cofilin-actin rods, and CIN knockdown under ATP depletion did not result in rod formation (Huang et al., 2008). This ATP-dependent increase in CIN activity is likely due to the disruption of the interaction between Hsp90 and CIN. Hsp90 binds and hydrolyzes ATP, and in addition to its function as a chaperone, it can also regulate CIN activity. Inhibition of Hsp90 alone can also induce cofilin-actin rod formation, suggesting that CIN bound to Hsp90 cannot dephosphorylate cofilin (Huang et al., 2008). As CIN is highly expressed in the brain and its activation leads to cofilin-actin rods, CIN could be a key contributor to AD pathologies.

## Summary and conclusion

Cytoskeletal dysregulation is closely linked to neurodegenerative disease. Key oxidative and energetic stress sensing pathways have been elucidated, and numerous methods for inducing the stress condition in cells have been identified. Cofilin-actin rods, Hirano bodies, and other aberrant cytoskeletal structures are the result of oxidative and energetic stress. Genetically-encoded methods for monitoring and mimicking these cytoskeletal elements are emerging; peptide, protein, and small molecule inhibitors of rod formation have been identified but have yet to reach the clinical stage. Finally, numerous proteins are involved in mediating cytoskeletal dysregulation; development of holistic models that tie protein networks to disease-based outcomes is an ongoing endeavor.

While the formation of cofilin-actin rods is only one strand in the web of pathways associated with dysregulation of the actin cytoskeleton, the inhibition or activation of cofilin-actin rod associated proteins may be important for rescuing synaptic loss and restoring cognitive ability in those affected by cognitive decline. Undoubtedly, unique strategies for the detection and regulation of cofilin-actin rods will continue to be developed, and new small molecules and peptides will emerge for cytoskeletal regulation. These developments are critical, as the need for effective therapies to combat neurodegenerative disease remains. Investigations into cofilin-actin rod formation and the neurological damage they incur can assist macro-scale studies of neurodegenerative diseases and provide causal connections amongst similar disease pathologies.

## Author contributions

All authors contributed to the writing of this manuscript. AW and RH generated the figures for this manuscript. AW, AS, CO’B, and RH conducted bibliographic research.

## Abbreviations

α-syn: alpha-synuclein
Aβ: amyloid Beta
AbpB: actin-bundling protein B
AD: Alzheimer’s disease
ADAM10: A disintegrin and metalloproteinase
Aip1: actin-interactin protein 1
AMPAR: AMPA receptor
APP: amyloid precursor protein
ASD: autism spectrum disorder
ATM: ataxia-telangiectasia mutated kinase
CAP: cyclase-associated protein
CorA: coronin
CuB: cucurbitacin B
Drebrin: drebrin
EB3: end-binding protein 3
Egr-1: early growth response-1
ET-1: endothelin-1
ET_*B*_: endothelin type B receptor
Fia: filactin
FTD: frontotemporal dementia
HD: Huntington’s disease
Hsp70: heat shock protein 70
HSV-1: herpes simplex virus type 1
LIMK: LIM kinase
LTD: long term depression
LTP: long term potentiation
MAPK: mitogen-activated protein kinase
MK2: MAPK-activated protein kinase 2
NOX: NADPH oxidase
PAK: p21-associated kinase
Pin1: peptidyl-prolyl *cis-trans* isomerase NIMA-interacting 1
PD: Parkinson’s disease
PrPc: cellular prion protein
PRRT2: proline-rich transmembrane protein 2
PTEN: phosphatase and tensin homolog
ROCK: Rho-associated coiled-coil protein kinase
ROS: reactive oxygen species
SCZ: schizophrenia
SGK1: serum- and glucocorticoid-inducible kinase 1
SMA: spinal muscular atrophy
SSH1: slingshot homolog-1.
